# The History of the Greek Refugee Hospital of the Interwar Period in a General Hospital of Nikaia “Agios Panteleimon”

**DOI:** 10.7759/cureus.54698

**Published:** 2024-02-22

**Authors:** Alexandra Mpakosi, Vasileios Cholevas, Ioannis Tzouvelekis, Stamatios Cholevas, Panagiotis Rafail Gavrilis, Areti Katsouda, Ioannis D Passos, Olympia Kalampaliki, Maria Mironidou-Tzouveleki

**Affiliations:** 1 Microbiology, General Hospital of Nikaia, Piraeus, GRC; 2 Medicine, University of Bologna, Bologna, ITA; 3 Agricultural Technology, Food Technology and Nutrition, Alexander Technological Educational Institute of Thessaloniki, Thessaloniki, GRC; 4 Animal Sciences, Agricultural University of Athens, Athens, GRC; 5 Medicine, Aristotle University of Thessaloniki, Thessaloniki, GRC; 6 Surgery, 219 Mobile Army Surgical Hospital, Didymoteichon, GRC

**Keywords:** american women's hospital, piraeus, asia minor catastrophe, interwar period, refugee hospital

## Abstract

The Asia Minor Catastrophe caused the uprooting of thousands of Greeks from Asia Minor and their arrival in Greece. Especially in the areas around Piraeus, there was a large settlement of refugee populations. During that period, a small hospital was created, the “American Women’s Hospital,” by an initiative of the “American Women’s Union,” with the aim of treating and caring for suffering refugees. Within a decade, the hospital expanded and became a general hospital. In 1934, after the departure of the “American Women’s Service” from Greece, it was renamed “Refugee Hospital of Nea Kokkinia,” and then a year later (1935), it was renamed again to “Protypo Laiko Iatreio” (Model Public Clinic). In 1939, the construction of a larger hospital in Nea Kokkinia began. During World War II, the hospital managed to respond to the difficult conditions of the period and was renamed “General Hospital of Piraeus, Saporta Warehouse Building.” After the war, in 1953, it was renamed to General Hospital of Piraeus “Queen Frederika.” In 1986, it was renamed to Regional General Hospital of Nikaia “Damon Vassileiou” in honor of the Professor of Medicine of the University of Athens Damon Vassileiou who was one of the greatest Greek doctors. In 2001, it was renamed again to its current name General Hospital of Nikaia “Agios Panteleimon,” becoming one of the largest hospitals in the Balkans.

## Introduction and background

The effects of World War I on the Greek economy included a drop in exports, a large reduction in foreign currency inflows, and speculation due to the war. In this situation, Greece undertook the 1919-1922 Asia Minor Campaign, exceeding its financial capabilities and leading to its national ruin and over-indebtedness. Even more, Greece had to accept and resettle 1.2 million refugees resulting from the Asia Minor Catastrophe. It was characteristic that, while in 1912 the population of Greece was 2.7 million, in 1928, it reached 6.2 million [1].

After the Asia Minor Catastrophe, thousands of refugees began to arrive at the port of Piraeus every day. All public places in Athens and Piraeus, such as streets, squares, and sheds, were occupied by hundreds of people. Their life in the first settlements was miserable [2-5]. Many people were crowded into wooden shacks without proper infrastructure, water, sewage, electricity, and even furniture, beds, or chairs. The roofs of the settlements were perforated, allowing the air and the rain to pass into their interior, and the toilets were communal, dirty, and sources of contamination [5]. According to newspapers at that time, the refugees were a public health problem by carrying microorganisms into the population and causing epidemics. Even more, the press emphasized the social dimension of the problem, arguing that the unsafe behaviors of the refugees put public safety at risk [6].

The United States government and several American charitable organizations (such as the American Red Cross, Near East Relief, the American Women's Hospitals, the Armenian Red Cross, and the Young Men's Christian Association) initiated actions to the care of refugees [7,8].

According to the American Women's Hospitals archives, the refugees suffered from malnutrition, typhus, smallpox, tuberculosis, dysentery, and trachoma, a childhood eye disease caused by Chlamydia trachomatis. The American Women's Hospitals Service was responsible for the creation, management, and financing of all the hospitals connected to the Near East Relief orphanages that had been transferred from Asia Minor to Greece. In fact, many Near East Relief workers had died of typhoid or smallpox during the forced relocation of the orphans to Greece. In addition, the doctors from the American Women’s Hospitals organization and the nurses from the American Red Cross established schools to train Greek nurses in modern medical practices [8].

The Greek state, with the help of the above international humanitarian organizations, proceeded with mass vaccinations and disinfections and established refugee hospitals. However, approximately 75,000 refugees have been estimated to have died within the first months of their arrival in Greece from diseases such as tuberculosis, typhus, smallpox, and malaria [9,10]. Nevertheless, according to the archives of the General Statistical Service of the Ministry of National Economy during the interwar period, in the period 1928-1937, a continuous increase in the country’s population had been reported, without a corresponding increase in deaths. It was characteristic that, in 1928, 1/5 of the Greek population consisted of refugees from Asia Minor. However, according to the above archives, serious endemic diseases were recorded, mostly in the countryside, where there were more deaths than in the cities mainly from respiratory diseases; incidents during pregnancy, childbirth, and puerperium; and infant and child diseases. In general, infectious diseases were the greatest threat to the public health of the Greek population of that period [11]. It was characteristic that, until 1936, the average life expectancy in Greece was 34 years. Meanwhile, in the United States, it was 65 years; in Sweden, 61 years; in Germany and Denmark, 60 years; in Norway, England, and the Netherlands, 55 years; in Austria and Switzerland, 54 years; in Poland, 46 years; and in Spain, 42 years. The Greek economy was also affected by this morbid situation of the citizens as there were many shortages of workers due to illness or even death [12]. According to the cause-of-death statistics records, in the period 1928-1937, most deaths were recorded as a result of infectious diseases: 193,883, of which 50,797 died from malaria and 99,445 from tuberculosis [11].

This situation forced the state to organize an integrated health system in order to effectively deal with the care of the country's population, which had grown steeply with the inclusion of the newly arrived refugees. Thus, during the interwar period, the state took over for the first time a leading role and not just an accessory to private initiative (as was the situation until then) for the health system organization (establishment of state sanatoriums and clinics, state hospitals, and the organization of the social insurance institution, which had also local health services). The interwar period, therefore, was a period in which many public health services such as hospitals were created [13].

Moreover, the Metaxas government proceeded to increase the number of beds in their existing hospitals by 1,180 beds. The total cost was approximately 48,200,000 drachmas, a significant amount given the difficult Greek economic situation. In addition, 54,750,000 drachmas were given for the construction of new buildings throughout Greece. Priority was given to the creation of new health units in areas where there was a great increase in population due to the arrival of refugees, such as in Kokkinia of Piraeus, where 5,000,000 drachmas were given to establish a hospital [12].

## Review

**Figure 1:**
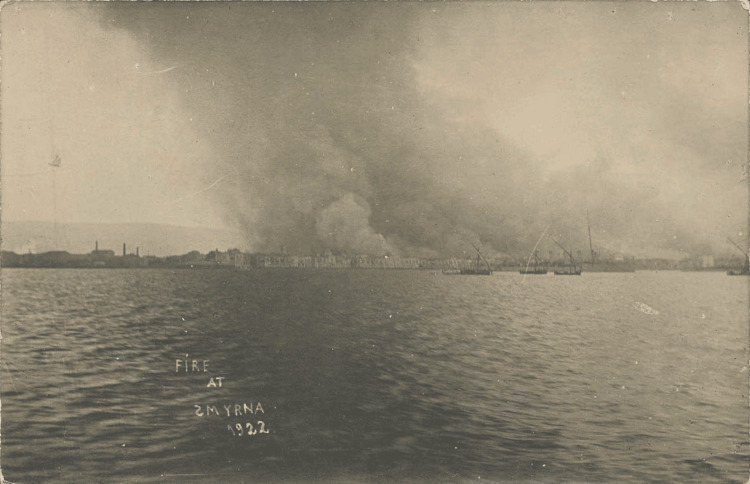
The great fire of Smyrna (1922) Source: Auction at karamitsos.com; public domain (Wikimedia Commons): https://commons.wikimedia.org/wiki/File:Smyrna_Fire_Sept_1922_from_HMS_Ajax.jpg

**Figure 2:**
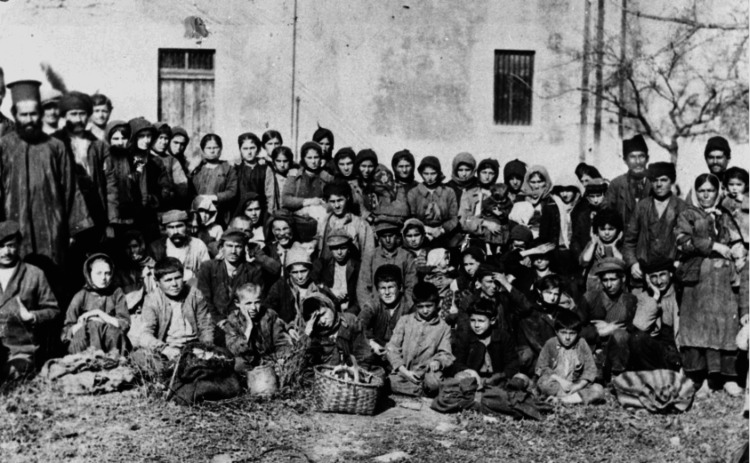
Refugees from Asia Minor Source: www.refugees-to-ionio1922.eu; public domain (Wikimedia Commons): https://upload.wikimedia.org/wikipedia/commons/0/0e/Pontian_Greek_refugees_from_Asia_Minor_Corfu_1923_01.jpg

**Figure 3:**
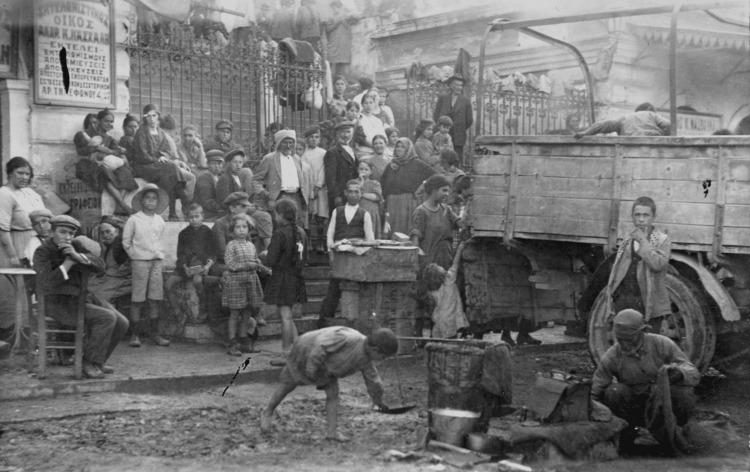
Refugee crisis Source: https://www.loc.gov/pictures/item/2010650538/; public domain (Wikimedia Commons): This image is available from the United States Library of Congress's Prints and Photographs division under the digital ID cph.3c39262.

**Figure 4:**
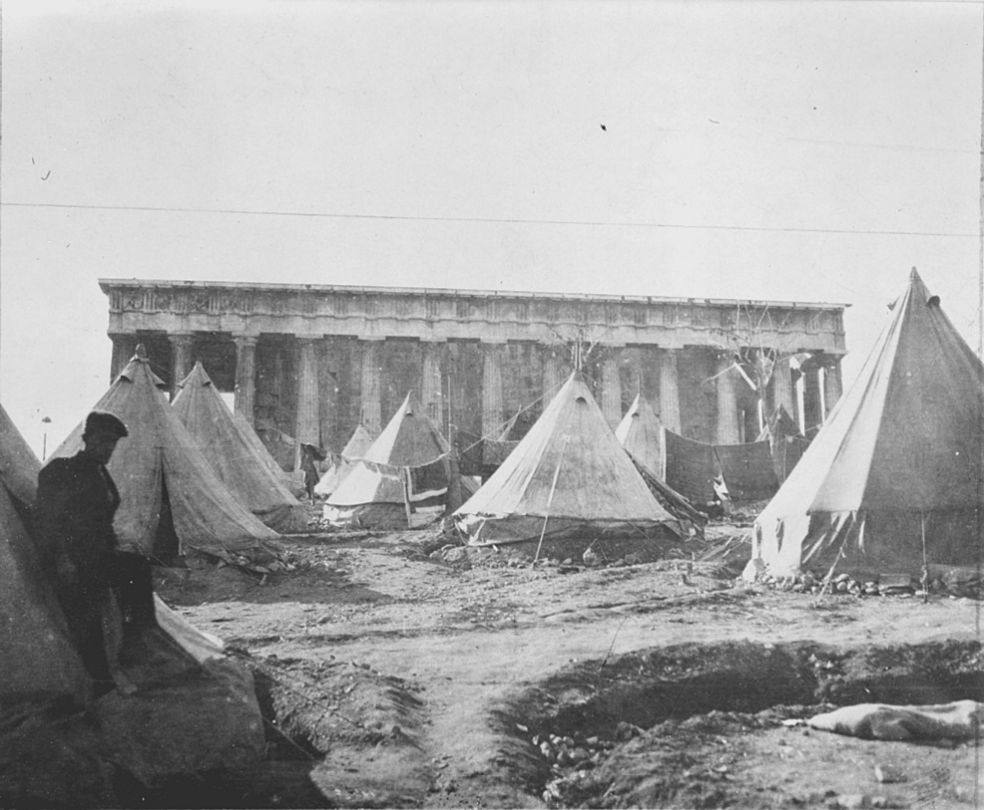
Makeshift refugee camps at the Temple of Theseus in Athens Source: https://www.loc.gov/pictures/item/2010650546/; public domain (Wikimedia Commons): This image is available from the United States Library of Congress's Prints and Photographs division under the digital ID cph.3c39254.

As mentioned above, in Greece, the end of World War I was followed by the Greco-Turkish War of 1919-1922, the Asia Minor Catastrophe, and the subsequent refugee crisis (Figures 1-4).

**Figure 5:**
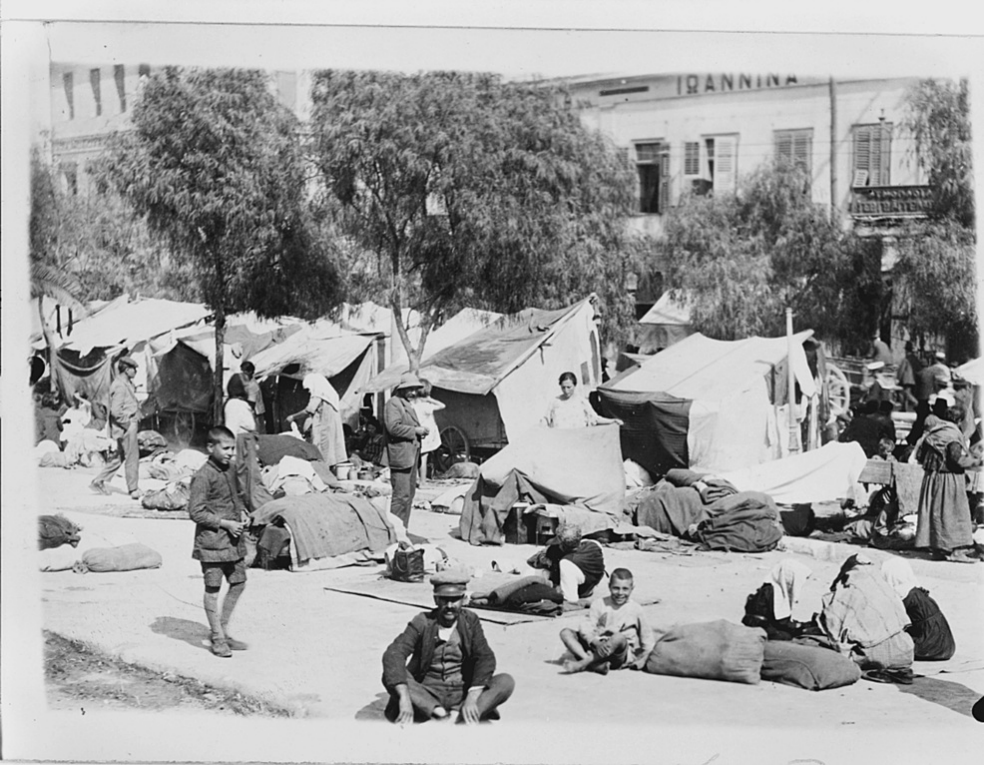
Tents placed in a square in Piraeus for the temporary accommodation of refugees from Asia Minor Source: https://www.loc.gov/pictures/item/2010650540/; public domain (Wikimedia Commons): This image is available from the United States Library of Congress's Prints and Photographs division under the digital ID cph.3c39260.

Piraeus, the country's largest port, received huge numbers of uprooted people. The entire coastal line of the port, the squares, and public places were filled with crude poorly made constructions with whatever materials anyone could find, such as discarded, broken wood, sacks, and sheet metal (Figure 5).

In 1922, by a government decision and with money from the Fund for Refugee Assistance (FRA), several plots of land were expropriated, and the refugee settlement of Nea Kokkinia was created, just 4 km from the center of Piraeus [14]. The name was due to the color of the soil of the area (kokkino in Greek means red), while it was called Nea (new) to distinguish it from the already existing Palea (old) Kokkinia. In 1934, it became a separate municipality (Municipality of Nea Kokkinia), which was later renamed to the Municipality of Nikaia, a name that came from the Asia Minor city of Nikaia, where in 325 B.C. the first Ecumenical Synod was convened by the Byzantine Emperor Constantine [15].

The only municipal hospital, Tzaneio, was not sufficient to provide any medical assistance to this ever-growing population of the city. The absence of another municipal or state hospital in Piraeus was covered by some private clinics, where many times the hospitalization and care were insufficient or even wrong. Thus, the state was forced to requisition various buildings in Piraeus, such as the hotels in Neo Faliro or the Hatzikyriakeio Orphanage, which was already mandated from the time of the Balkan Wars and operated as a Military Hospital for a long time. Nevertheless, the continuous increase in the refugee population with the creation of additional refugee settlements around the city of Piraeus could not be covered even after the addition of the Hatzikyriakeio Orphanage, which was now named Hatzikyriakeio Refugee Hospital.

In 1924, a group of women from America, working for the American Women's Hospital Service, arrived in Piraeus and settled in a building granted to them by the Refugee Rehabilitation Committee, located in the area between Palaia and Nea Kokkinia, and began to offer charity work and medical care. As it has been reported, in Nea Kokkinia, the refugee population has reached then approximately 80,000 people in 7 km^2^. However, these American ladies contributed significantly to the medical care of those uprooted people, offering large sums of money for this purpose. Their building was called “American Hospital” or otherwise “American Women’s Hospital” [15]. The capacity of the “American Hospital” gradually reached 74 beds, most of which were intended for women, who worked under harsh conditions in the factories of the region.

**Figure 6:**
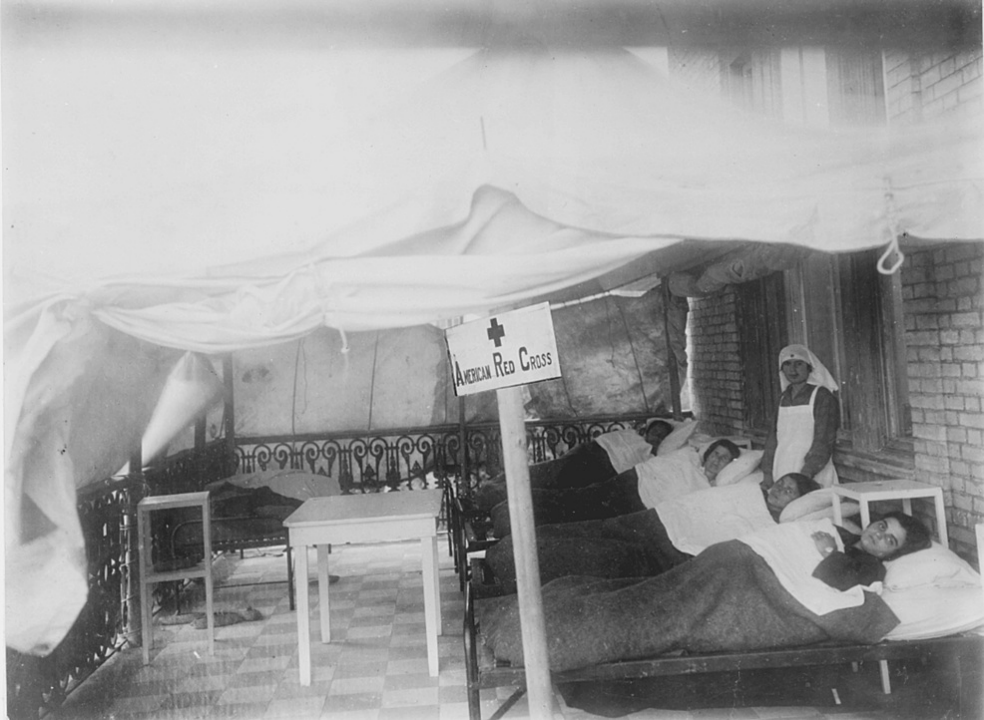
The Red Cross had wards supervised by the American Women’s Hospitals for the purpose of isolating any case of typhus and other contagious diseases Source: https://www.loc.gov/pictures/item/2010650521/; public domain (Wikimedia Commons): This image is available from the United States Library of Congress’s Prints and Photographs division under the digital ID cph.3c39329.

**Figure 7:**
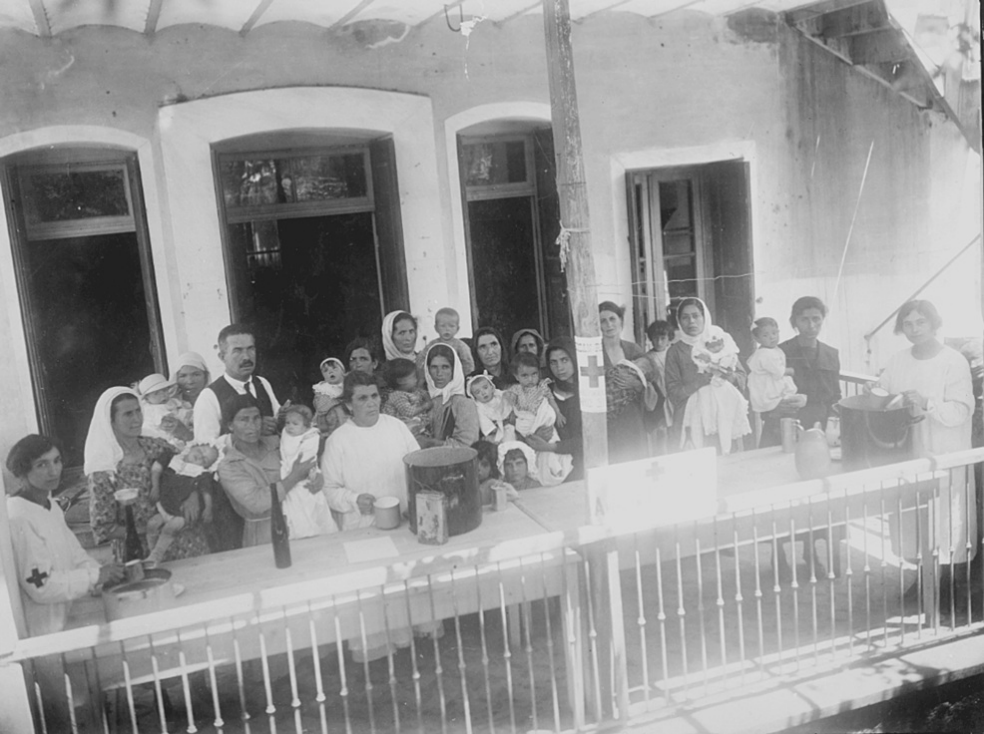
Red Cross milk stations saved the lives of thousands of babies Source: https://www.loc.gov/pictures/item/2010650523/; public domain (Wikimedia Commons): This image is available from the United States Library of Congress’s Prints and Photographs division under the digital ID cph.3c39331.

The contribution of the United States government and American charitable organizations (the American Red Cross, Near East Relief, American Women's Hospitals, the Armenian Red Cross, and the Young Men's Christian Association) was decisive for the care of refugees [7,8] (Figures 6-7).

**Figure 8:**
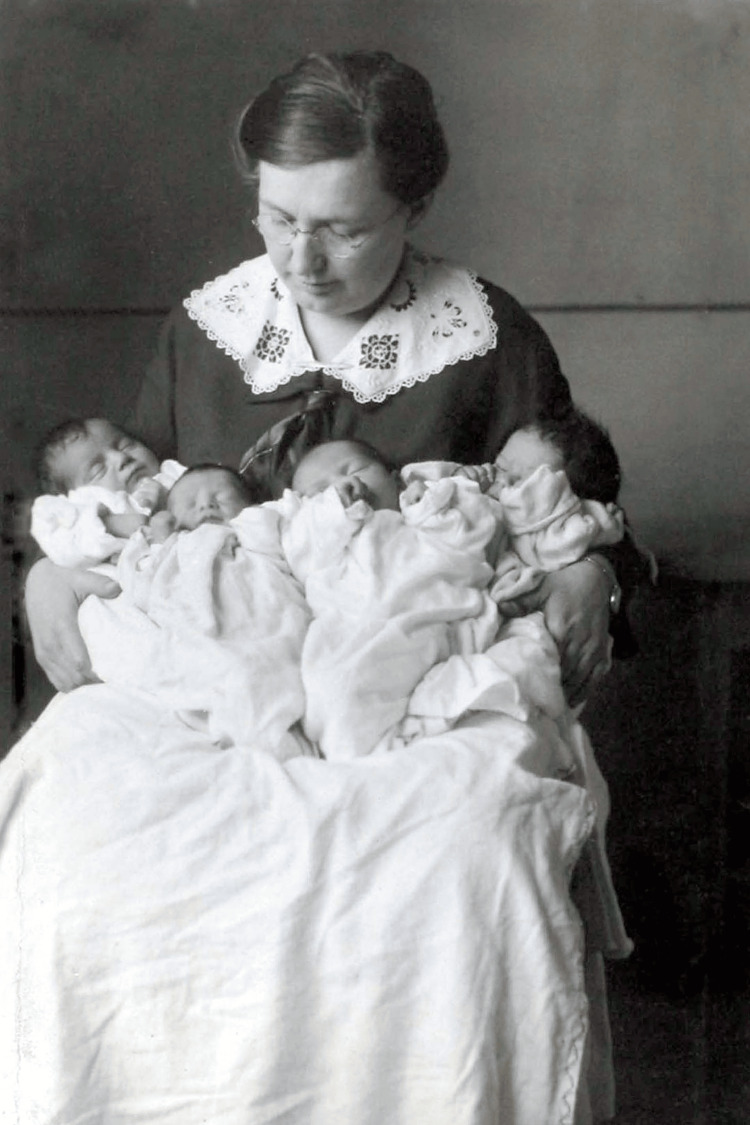
Dr. Ruth Azneve Parmelee Source: http://www.dlir.org/archive/orc-exhibit/items/show/collection/8/id/15376; Creative Commons Attribution 3.0 (Wikimedia Commons)

The American Hospital in Nea Kokkinia was under the supervision and control of the American Women's Union, which, during World War, I had established similar hospitals in France, Serbia, and Russia. The American Women’s Hospital was also financed by donations from the various crafts that had been established in the refugee area, due to the cheap labor force, as well as by donations from Greeks and Philhellenic Americans. These financial donations were managed by the American Mrs. Edison. The staff of the “American Hospital” consisted of Dr. Ruth Parmelee, who was the director and had come to Greece as a member of the charity mission; the head nurse Emilia Williams; and a number of Greek doctors who volunteered their services both inpatients and outpatients (Figure 8).

Dr. Ruth Azneve Parmelee (1885-1973) was a medical missionary born in Trabzon, Turkey, to missionary parents (her father, Dr. Moses Payson Parmelee, was a Christian missionary for the American Board of Commissioners for Foreign Missions, ABCFM). At the age of 11, she traveled to the United States where, in 1907, she received her bachelor's degree from Oberlin College and, in 1912, her master's degree from the University of Illinois [16]. In 1914, Dr. Parmelee was sent by the ABCFM to Turkey where she established a dispensary in Harput for the purpose of obstetrics and orphan care and the training of nurses. She continued this work until 1917 and for the period 1919-1922. In 1922, Dr. Parmelee, with two colleagues Dr. Mark H. Ward and Isabelle Harley, were deported from Turkey. In October 1922, she went to Thessaloniki, Greece, to help the Christians who had been expelled from Turkey by the Kemalists. There, she directed the medical assistance for the American Women’s Hospitals (AWH) and organized with other refugee doctors and nurses, camp services, clinics, a 100-bed hospital, and the training of nurses [17]. From 1925 to 1933, she was the director of both the AWH hospital in Nea Kokkinia (now called Nikaia), as well as the nursing school that had been transferred with her from Thessaloniki. Dr. Parmelee stayed and worked in Greece until 1941 when she left due to the German occupation. Greece owes her gratitude for her work both during the refugee crisis and in the post-war years when she returned to Athens offering her services: Between 1946 and 1947, she was a medical consultant and director of the School of Physical Therapy of the Near East Foundation, and between 1948 and 1953, she taught hygiene, community health, and medical information to social workers at Pierce College near Athens. She died in the USA in 1973. Her work was awarded in Greece several times: In 1924, King George II of Greece awarded her the Silver Cross of the Chevaliers of our Order of the Savior, while in 1953, King Paul appointed her the Order of Beneficence (Tagma tis Efpiias) [18].

Medical care in the period of the American ladies was exemplary: every two patients were cared for day and night by a nurse, while the hospital storerooms were filled with medical drugs, sheets, blankets, and other useful items [19].

After the departure of the American Women’s Hospitals Service from Greece, the hospital was transferred to the Greek state and renamed to “Refugee Hospital of Nea Kokkinia.” A brotherhood was established by elite members of the Piraeus and Nea Kokkinia communities, and the former “American hospital” was then placed under the control of the Nea Kokkinia Municipality and was renamed again to “Protypo Laiko Iatreio” (Model Public Clinic). However, the original goal of the brotherhood was to create a new modern building in place of the old one. However, the problems that had to be faced were many. Nea Kokkinia was then a poor area with unpaved and dirt roads and no sewage network. Additionally, the building that housed the former “American Women’s Hospital” was very small, and the financial resources were minimal [19]. Thus, the brotherhood simply converted the “American Women’s Hospital” into the “Protypo Laiko Iatreio” (Model Public Clinic), which operated for about four years. In 1938, the government of Prime Minister Metaxas closed the refugee hospital in Hatzikyriakeio, which has since reopened in its original form as an orphanage, and decided to create a second hospital in the Piraeus region. Decisive in this decision taken by the government was the long-standing request of the brotherhood for the construction of a new and larger hospital in Nea Kokkinia. Metaxas then asked the Municipality of Piraeus to grant for this purpose an additional area on the borders of Palaia Kokkinia and Nea Kokkinia. Thus, in 1939, land belonging to the church of Agii Anargyri was granted, and the construction of the Nea Kokkinia hospital began. Reinforced concrete was used entirely for its construction. In 1940, Nea Kokkinia was renamed to Nikaia [19,20].

By October 1940, when World War II began, the basement, ground floor, and two floors of the west wing had already been built of reinforced concrete. The Germans, from the very first period of their occupation, were impressed by the solidity and safety of construction offered by reinforced concrete, which distinguished the hospital from other buildings in Greece. Thus, they turned it into warehouses for their army material. In the meantime, since the beginning of the war, the brotherhood had moved, for security reasons, the former “American Women’s Hospital” to the tobacco warehouse buildings nearby. The full name of the hospital at that time was “General Hospital of Piraeus, Saporta Warehouse Building.” Saporta was the name of the family of tobacco manufacturers in whose warehouses the hospital was housed. During the war, the hospital managed to respond to the adverse conditions of the period, as well as the urgent and regular health needs of the population [20]. In 1941, the illegal resistance organization “National Solidarity of Piraeus” was founded with the aim of collecting money for the relief of the war invalids who were being treated in the Tzaneio and Saporta hospitals. “National Solidarity Piraeus” also helped the tuberculous resistance fighters, as well as the families of the executed or imprisoned resistance fighters through contacts and links that had been created within the Saporta hospital. It stayed in the tobacco warehouse buildings until 1952 when it was transferred again to the new facilities of the hospital in its current location [19].

The end of the war found Greece financially devastated, while a worse war followed, the civil war. Since the period of the German Occupation, the management of the country's hospitals had passed from the control of the municipalities to the control of the state. This situation continued even after the end of the war, with the result that the hospitals that had been built and maintained for years by donations from the residents came under the control of the bankrupt state [19].

**Figure 9:**
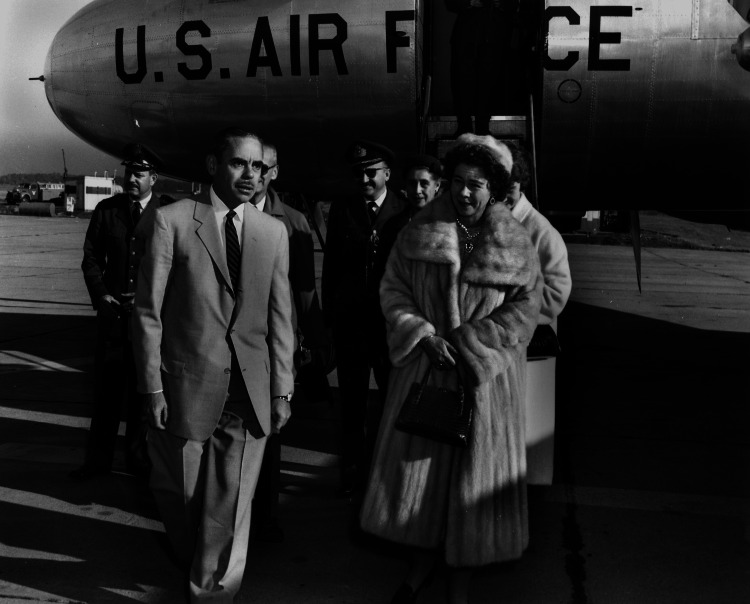
Queen Frederika of Greece Source: Queen Frederika - Queen consort of the Hellenes arrival at Knoxville's McGhee Tyson Airport; public domain (Wikimedia Commons)

Therefore, for the subsequent reconstruction of the hospital, the brotherhood requested a loan to cover part of the required amount from the Real Estate Bank. For the rest, a “Pampiraic Fundraiser” under the supervision of the queen of Greece Queen Frederika was carried out (Figure 9). However, all the money collected was not enough to rebuild the hospital.

In 1947, the American government provided the financial assistance needed to complete the first and second wings of the hospital. In addition, the Americans allocated 350 thousand dollars for the equipment of the hospital. The head of the American mission in Greece, Mr. Burroughs, personally supervised the work. The American financial aid was clearly related to the institution's history as an “American Women's Hospital.”

The hospital was named “Queen Frederika.” On January 26, 1953, the General Hospital of Piraeus “Queen Frederika” was inaugurated by the Queen herself. It was decided to bring the title of “Piraeus” instead of “Nikaia” due to the area granted by the Municipality of Piraeus but also due to the “Pampiraikos fundraiser” [19].

**Figure 10:**
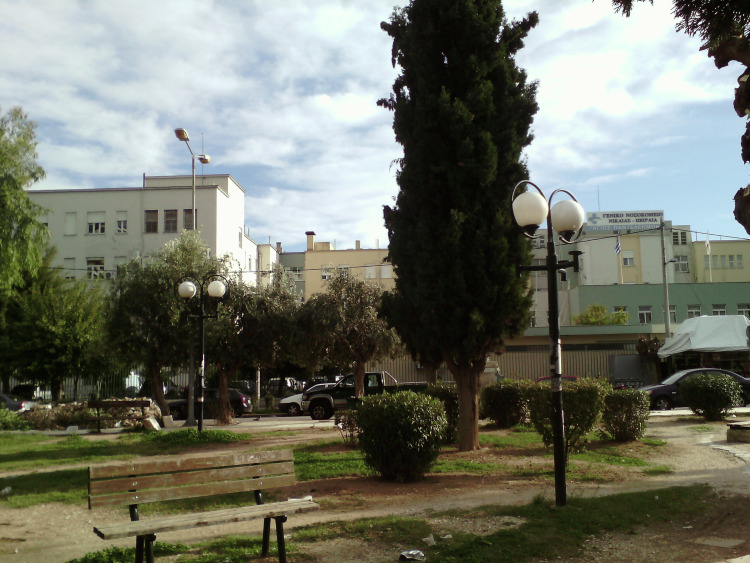
General Hospital of Nikaia “Agios Panteleimon” Creative Commons Zero, Public Domain Dedication (Wikimedia Commons)

The General Hospital of Piraeus began to operate normally, steadily expanding. His medical service included pathological and surgical departments. In addition to these medical departments, the organization of the hospital provided for the operation of outpatient clinics and laboratories. From 1955 to 1956, it reached 400 beds, while from 1954, it was strengthened with a Center for Blood Donation and Preparation of Blood Derivatives. In 1964, the hospital had 600 beds and all the necessary laboratories. In 1986, it was renamed to Regional General Hospital of Nikaia “Damon Vassileiou” in honor of the Professor of Medicine at the University of Athens Damon Vassileiou, who was one of the greatest Greek doctors. Its beds then reached 630. In 1995, it had 716 beds, of which 343 in the Pathological, 343 in the Surgical, and 30 in the Psychiatric Department. In 2001, it was renamed to its current name General Hospital of Nikaia “Agios Panteleimon” with a church of the same name (“Agios Panteleimon”) in its forecourt [20] (Figure 10).

## Conclusions

The interwar era was a particularly turbulent period for Greece. The Asia Minor Catastrophe caused the arrival of thousands of refugees and the increase of the country's population. The accumulation of these uprooted people in makeshift, unsanitary camps increased disease rates. The Greek state, with the help of foreign humanitarian organizations, tried to deal with this situation by establishing hospitals specifically for refugees. One of them was the General Hospital of Nikaia “Agios Panteleimon,” which was created for this purpose in the refugee area of Nea Kokkinia (now called Nikaia) and has withstood the passage of time, becoming today one of the largest hospitals in the Balkans.
